# Letter to the Editor

**DOI:** 10.2188/jea.12.341

**Published:** 2007-11-30

**Authors:** Osman Mansoor, David Bassett, Jeffrey McFarland, Yang Baoping, Shigeru Omi, Chushi Kuroiwa

**Affiliations:** 1 World Health Organization, Regional Office for the Western Pacific; 2 Department of Health Policy and Planning, School of International Health, Graduate School of Medicine, University of Tokyo

##  

Dear Sir,

In a recent article (J Epidemiol 2001; 11: 255-262), flawed analysis of limited data led to an incorrect conclusion that a measles immunization campaign in the Lao People’s Democratic Republic had minimal impact. The article also inaccurately portrays WHO policy.

On October 29, 2000, the Western Pacific Region was certified as polio-free.^1^ Laos’ historic achievement in eliminating polio was questioned at that time by the same authors.^2^ But, Laos remains polio-free.

Despite the cynics and the nay-sayers, global smallpox eradication and regional polio elimination were achieved. The paper rightly points out that measles eradication will be even harder and that measles control efforts must not interfere with polio eradication. However, that is not the issue for Laos, where polio has been eliminated and a high standard of acute flaccid paralysis surveillance is being maintained.

A proven and extremely cost-effective way of rapidly controlling measles in countries with poor routine immunization coverage is a measles immunization campaign.^3^^,^^4^^,^^5^^,^^6^ Ignoring this evidence the paper concludes that “our data showed the limited outcomes of mass vaccination campaign.”

The conclusion is based on an estimate of vaccine efficacy (VE) in a small number of outbreaks before the campaign. Outbreaks, by their nature, lead to lower VE estimates.^7^ The VE estimate of 68% is used to arbitrarily guess that protection after a second dose would only be 80% effective. In fact, two doses of measles vaccine are about 99% effective.^8^^,^^9^ The article uses the VE estimates multiplied by estimated coverage to compare percentage immunity with and without a campaign. The difference in immunity is then multiplied by the percent of cases vaccinated to estimate the percent reduction in measles from the campaign. This calculation not only fails to appreciate the indirect effect of immunization (‘herd immunity’) but also that the proportion of cases who are immunized increases with immunization coverage. The article then further minimizes the estimate of impact of the campaign by multiplying the percent reduction to a total number of reported cases for a year during which there were relatively few cases. There is no consideration that most measles cases are not even reported.

In addition to the flaws in logic and mathematics, the VE estimate deserves further scrutiny. Simply providing a statistical confidence interval to the point estimate shows that there is a 95% chance that the VE could be up to 79%. More important than the play of chance are the biases of the screening method. To start with, there is always uncertainty about the true immunization status of the cases, and the investigators do not state whether they accepted a history without written documentation. A sub-analysis for cases with vaccination cards is likely to show higher VE.

Reported coverage figures are known to be somewhat unreliable. The use of national coverage data for these populations introduces another bias. The fact that these were the sub-set of outbreaks in locations that could be reached by the investigators suggests that coverage in these populations is likely to be higher than the national average. The higher the coverage, the greater the proportion of cases that will be immunized.

The WHO strategy is not, as suggested by the authors, for the initial immunization campaign to target only children up to the age of five years. However,this age was recommended in Laos because of the difficulties highlighted by the authors, to test whether any sort of measles immunization campaign could be undertaken, and to protect the many children who remained unprotected because of previous low routine immunization coverage. In other countries in the Region, measles campaigns have targeted older children, depending on local circumstances and epidemiology.

Accelerated measles control requires two doses of measles vaccine, not the three suggested by the authors. This is because the follow-up campaign targets those who missed out on the previous campaign to ensure that every child has a second opportunity. Another way to improve measles control is to offer two doses in the routine immunization schedule. This method for measles control will take longer than can be achieved through a well-coordinated campaign.

In conclusion, the paper reflects a poor understanding of WHO policy and indeed of measles epidemiology. Limited data are manipulated to make a case that is not in line with actual country experience. Measles immunization campaigns are an important and effective way of decreasing measles morbidity and mortality.

Osman Mansoor, David Bassett, Jeffrey McFarland, Yang Baoping, and Shigeru Omi

World Health Organization, Regional Office for the Western Pacific, United Nations Avenue, P.O. Box 2932, 1000 Manila, Philippines.

(Received April 15, 2002, and accepted April 18, 2002)

## In Reply

Dear Sir,

Mansoor et al. of WHO regional office for western pacific region (WPRO) raised two major comments on our article.^1^ The first comment refers to the validity of our data with a small sample size, and the second one is about the calculation of vaccine efficacy. We acknowledge that there were several limitations in our study, yet it must be noted that it was the first study in Laos that examined the feasibility of measles elimination programme, using the number of confirmed measles cases diagnosed by IgM, together with the real vaccine history. In our latest study where 230 blood samples of children aged 9 months to 4 years were measured for measles antibodies before and after the pilot measles vaccination campaignin in 2000,^2^ we tried to overcome these limitations. This study explored that the prevalence of measles antibody of children with two doses increased to 94% (lower than 99% that Mansoor et al. referred), and the increase of the antibody among the target population after the campaign was 24%, which is close to the prediction of 22% in our initial study.^1^ Another main finding of this study was that seroconversion rate by the campaign was only 74% at the community level.^2^ All what we wanted to do was to obtain the data as reliable as possible and to examine the feasibility of measles elimination programme in Laos, because the program was thought to be implemented just following the experiences of Pan American Health Organization (PAHO), without reliable baseline data in Laos.

Besides, the letter also mentioned about our earlier report on polio eradication in Laos.^3^ We must say, however, that they misunderstood our message in that report, where in fact we tried to emphasize the importance of putting more efforts towards surveillance for acute flaccid paralysis, and of maintaining high coverage with oral polio vaccine even after the regional declaration for eradicating poliomyelitis in the western pacific region. Although they claims that Laos has the high standard of acute flaccid paralysis surveillance, our another survey in Laos conducted from November 2000 to June 2001 revealed that there were 15 acute flaccid paralysis cases unreported, of which 6 were found in November 2000 just one month after the declaration (unpublished data).

Either it was on measles control or on polio eradication, the messages proposed in our papers^1^^-^^3^ were based on the experience through the projects by the Japan International Cooperation Agency (JICA) since 1992. I was involved as an EPI expert in Primary Health Care (PHC) project (1992-1996) and as a chief adviser in Pediatric Infectious Disease Prevention (PIDP) project (1998-2001), under the collaboration with Ministry of Health (MoH) in Laos. One of the main component of PHC project was to support the expanded programme on immunization (EPI), where the project successfully established high quality of AFP surveillance system for polio eradication by conducting frequent and intensive field visits at community level.^4^ Likewise, the PIDP project also aimed to ensure the achievement of the polio eradication as well as to establish the good surveillance system for vaccine preventable diseases. Throughout these two projects, there were biweekly epidemiology meeting held with the surveillance unit of MoH and WHO, and the interagency coordinating committee meeting on EPI composed of MoH, JICA, WHO, and UNICEF. These meetings indeed enabled us to share all the relevant data each other. One of our common goals was to seek for more appropriate measles control programme. Although we had once submitted the draft^1^ to WPRO on March 9, 2001, no comment was given at that time, and the letter by Mansoor et al. was indeed the first one we received.

In response to their remarks on our criticism regarding the measles campaign, we would like to point out that, in 2002, there was a big outbreak in Phongsaly and Vientiane province where the campaign was conducted in 2000 and 2001 respectively. Six hundred and thirty-six measles cases (43% children were under 5 years old) including 11 deaths were reported in Phongsaly, and 219 cases (74% under 5 years) were reported in Vientiane. Besides, the letter mentioned that the control would require only two doses, instead of three doses as we had suggested. Yet, the third dose means the follow-up campaign, which has to be repeated every year together with routine immunization in order to interrupt virus transmission. Hence, the children have to receive three times vaccines until they become 5 years old; (1) routine immunization, (2) initial campaign, and (3) follow-up campaign. In addition, there are a couple of discrepancies between the comments by Mansoor et al. and the descriptions in the guideline by WPRO.^5^ For example, the letter says as if Laos is the only country with the campaign targeting under 5-year-old children only, though there are also other countries (e.g. Cambodia, Papua New Guinea, China, and Mongolia), according to the regional plan for immunization activities for accelerated measles control 1998-2003 in the guideline.

Needless to say, in order to implement global infectious disease control, it is crucial to take various opinions from the government, as well as local staff of international and non-governmental organizations. We have been concerned that the global infectious disease control programme should not be implemented as top-down policy. We must appreciate the ability of the local staff who know the actual situations, so that the country-specific policy can be successful. Otherwise, global health policy can be just activities without feasible goal.

Chushi Kuroiwa

Department of Health Policy and Planning, School of International Health, Graduate School of Medicine, University of Tokyo, 7-3-1 Kongo, Bunkyo-ku, Tokyo 113-0033, Japan.

(Received July 1, 2002, and accepted July 3, 2002)
